# The effect of orthokeratology lenses on optical quality and visual function in children

**DOI:** 10.3389/fnins.2023.1142524

**Published:** 2023-04-14

**Authors:** Weiwei Lu, Guanxin Song, Yuhan Zhang, Yan Lian, Ke Ma, Qingqing Lu, Yiyu Jin, Yang Zhao, Shuyu Zhang, Fan Lv, Wanqing Jin

**Affiliations:** ^1^State Key Laboratory of Ophthalmology, Optometry and Visual Science, Eye Hospital, Wenzhou Medical University, Wenzhou, China; ^2^Department of Ophthalmology, The Fourth Affiliated Hospital of China Medical University, Shenyang, China

**Keywords:** orthokeratology lenses, children, visual function, optical, quality

## Abstract

**Purpose:**

To assess changes in optical quality and visual function in children after 3 months of wearing orthokeratology (OK) lenses.

**Methods:**

A total of 25 myopic children aged 8–12 years were recruited and completed the follow-up study. Optical quality, visual function and corneal morphology were assessed at baseline and at follow-ups 1 and 3 months after wearing OK lenses. Optical quality parameters mainly included the modulation transfer function (MTF) cutoff, objective scattering index (OSI), Strehl ratio (SR) and the predicted visual acuities (PVAs). Visual function was assessed by visual acuity, monocular contrast sensitivity function (CSF) across five spatial frequencies and the area under the log contrast sensitivity function (AULCSF) that was also computed as an index for overall CSF.

**Results:**

The MTF cutoff and SR values both increased after 1 month of wearing the OK lenses (baseline vs. 1 month: *P*_MTF_ = 0.008 and *P*_SR_ = 0.049); this improvement plateaued after 3 months of lens wear (1 month vs. 3 months: *P*_MTF_ = 0.626, *P*_SR_ = 0.428). The corneal morphology also showed the similar change trend. The OSI showed the opposite change trend (baseline vs. 1 month: *P*_OSI_ < 0.001; 1 month vs. 3 months: *P*_OSI_ = 0.720). The mean CSF at 1.5 cpd decreased significantly after 1 month of wearing the lenses (baseline vs. 1 month: *p* = 0.001) and recovered after 3 months of lens wear (baseline vs. 3 months: *p* = 0.076). CSF at spatial frequencies of 3, 6, 12 and 18 cpd as well as the AULCSF did not significantly differ between any two timepoints (all *Ps* > 0.05).

**Conclusion:**

After 3 months of wearing OK lenses, the subjects exhibited a decrease in optical quality, similar to corneal morphology, whereas their visual function remained largely unchanged. Thus, the optical quality was more susceptible to OK lenses than visual function in children. The initial month of OK treatment of children is a key period to be paid close attention to deterioration of optical quality and visual function.

## Introduction

The prevalence of myopia worldwide is increasing, impacting public health and raising serious concerns, especially in East Asia (Wu et al., 2016). Orthokeratology (OK) lenses, a special rigid contact lens with an inverse geometric design on the posterior surface, can flatten the central cornea and steepen the mid-peripheral cornea when worn at night. OK lenses effectively and securely delay 43–63% of axial length (AL) elongation and slow myopia progression, as demonstrated by most clinical medical evidence and reviews (Si et al., 2015; Sun et al., 2015; Hiraoka, 2022). In recent years, the proportion of myopic adolescents has increased, and OK lenses have become an increasingly popular treatment method in young populations for controlling myopia (Xie and Guo, 2016; Hiraoka, 2022). However, previous studies have found that the change in corneal morphology induced by wearing OK lenses leads to a rapid increase in optical scattering of light and aberrations (Santolaria-Sanz et al., 2015, 2016). The stability of the tear film (Xie and Wei, 2023) also affects the optical quality and visual function of children with OK lenses. Pupil size, the diameter of the treatment zone and OK lens decentration also have a potential influence on visual performance and optical quality (Faria-Ribeiro et al., 2016; Liu et al., 2018). These factors may lead to visual discomfort related to the impaired optical quality and visual function, such as glare, ghosting and distortion (Zheng et al., 2019). Therefore, investigating comprehensive changes in optical quality and visual function after wearing OK lenses is greatly important.

To our knowledge, most previous studies on optical quality and visual function involving OK lens treatment have been conducted with adults (Santolaria-Sanz et al., 2015, 2016; Tian et al., 2018). The results showed some deterioration in optical quality and visual function. However, at present, the majority of people wearing OK lenses are adolescents, especially in China. Therefore, the optical quality and visual function of adolescents needs more attention. However, few studies have examined the optical quality and visual function of children. Studies on children’s optical quality, such as ocular HOAs, corneal HOAs and the OSI, have shown a certain amount of deterioration after wearing OK lenses and no recovery after 1 year of wearing OK lenses (Guo et al., 2018; Liu et al., 2018; Singh et al., 2020). Regarding visual function, Singh et al. (2020) found no decrease in CSF after 1 month of OK lens wear. However, other studies have found that CSF declines after OK lenses, using a different assessment device (Chang and Cheng, 2020). Therefore, it is debatable whether CSF is affected by OK lens wear in children. This study aimed to determine whether objective and subjective factors are affected by OK lens wear in children.

Most related studies focused on the relatively one-sided parameters of optical quality and visual function or had a short follow-up period. Notably, there is a lack of comprehensive evidence with a longer follow-up periods, leading to an absence of clarity regarding the effect of OK lens wear on optical quality and visual function in children. Therefore, a prospective, self-controlled study was conducted to explore changes in optical quality, visual function and corneal morphology in myopic children after wearing OK lenses.

## Methods

### Participants

This study was a prospective and self-controlled clinical trial. From October 2020 to October 2021, a total of 33 myopic children (18 girls and 15 boys) who were willing to wear OK lenses were recruited to participate in the trial at the optometry clinic of the Affiliated Eye Hospital of Wenzhou Medical University. The inclusion criteria were age of 8–14 years, spherical refraction between −0.75 D and −5.00 D, astigmatism <1.50 D, and best-corrected visual acuity of 0.1 logMAR or better. The exclusion criteria were as follows: the presence of ophthalmic disease, strabismus, a history of ocular surgery, obvious lens decentration (>1 mm based on corneal topography) during follow-up, and a history of contact lens wear. During the 3-month follow-up, eight subjects were excluded from this study due to failure to complete follow-up examinations (because of COVID-19) or obvious lens decentration (>1 mm based on corneal topography) for 3 months. Ultimately, 25 subjects (13 girls and 12 boys), aged 10.48 ± 1.18 years (range: 8–12), completed all follow-ups. The mean spherical refraction was −2.53 ± 0.73 D (range: −1.25 D to −4.25 D), and the mean astigmatism was −0.31 ± 0.33 D (range: 0.00 D to −1.50 D).

All participants and their parents signed consent forms after the purpose, procedures, and possible risks of the study were explained to them. This study was approved by the Ethics Committee of the Eye Hospital of Wenzhou Medical University (2019077K76). The study design was in accordance with the WHO’s definition of a clinical trial, as stipulated in the Declaration of Helsinki. The clinical registration code was ChiCTR1800019893.^1^

### Study procedures

OK lenses are a special type of rigid contact lens with an inverse geometric design that can correct low to moderate myopic refraction and slow axial length elongation. The brand of OK lenses used in this study was Dreamlite (Procornea Nederland B. V Corporation, Netherlands), manufactured with Boston XO (DK: 100 × 10-11[cm^2^/s][mL O_2_/mL·mmHg]) with a four-zone reverse geometric design. The lens consisted of a central base curve with a 6.0-mm optic zone diameter, a 0.6-mm wide reverse curve, a 0.9-mm wide alignment curve, a 0.4-mm wide peripheral curve and a Jessen factor of 0.75. The central thickness was 0.22 mm.

Before fitting the OK lens, the ocular health of subjects was evaluated, including the quality of tear film, condition of cornea and conjunctiva, uncorrected visual acuity (UCVA), refraction, intraocular pressure, corneal topography, fundus photography and axial length. Lens fitting evaluation was performed according to the manufacturer’s fitting guidelines. Well-fitting lenses had a classic bullseye fluorescein pattern, between 1.0 mm and 1.5 mm of lens movement, were centered on the cornea, and typical corneal topography with no obvious lens decentration (>1 mm). The participants were instructed to wear the OK lenses for 8–10 h per night. All subjects were followed up at the second day, the first week, the first month and the third month of OK lens wear. Afterward, OK lens wearers were advised to visit every 3 months. The follow-up examination included common follow-up items (evaluation of ocular surface safety, lens fit, UCVA, and corneal topography) and special items (Optical Quality Analysis System™ II (OQAS^II^) and CSF). Optical quality and visual function was evaluated only at baseline, 1-month and 3-month follow-ups. The vision-related questionaires regarding uncomfortable experiences (such as glare, holes, distortions, asterism, scieropia, and blurred vision) was administered at follow-up.

In this study, **UCVA,** corneal topography, optical quality and visual function before and after wearing OK lenses were compared. Corneal topography (Medmont E300, Medmont International Pty, Australia) was used to measure the shape of the cornea at least three times in every eye. Steep K and flat K values (simulated keratometry readings at the flattest and steepest meridians, respectively) in corneal topography before and after OK lens wear were obtained. UCVA was measured by the standard logarithmic visual acuity chart.

### Optical quality

The optical quality of subjects was assessed by a double-pass optical quality-Optical Quality Analysis System™ II (OQAS^II^; Visiomereics SL, Terrassa, Barcelona, Spain), which can comprehensively quantify light scatter and optical aberrations and measure the overall objective optical quality of the eyes (Xu et al., 2015; Henriksen et al., 2016). A single light source was produced by a 780-nm laser beam adequately filtered and collimated. The beam image was projected onto the eye and reflected on the retina. The light crossed the ocular medium twice. OQAS analyses the size and shape of the reflected point of light (Xu et al., 2015). Details about this measurement system are available in Güell et al. (2004).

OQAS II yields six main parameters: (1) OSI, which objectively and completely quantifies the intraocular scattered light representing the scattering index of refractive media, such as the cornea, lens and vitreous humor; (2) the modulation transfer function cut off frequency (MTF cutoff), which is the ratio of contrast between the retinal image and the original image determined by OQAS II, with the cutoff reflecting the cutoff frequency at 1% of the maximum MTF (Artal et al., 2011; Wang et al., 2016); (3) the Strehl ratio (SR), which is the ratio of the area under the MTF between the measured eye and the ideal eye; and (4) predicted visual acuities at contrast levels of 100%, 20%, and 9% (PVA100%, PVA 20% and PVA 9%), which were defined as the predicted best visual acuity at 100%, 20%, and 9% common contrast conditions in OQAS^II^ (Xu et al., 2015). Higher MTF cutoff, SR, PVA100%, PVA 20% and PVA 9% values and lower OSI values indicated better optical quality.

The reliability of this instrument has been confirmed in previous studies (Kamiya et al., 2012; Iijima et al., 2015; Leung et al., 2017). To ensure effective and accurate quantification of the image quality of the retina using OQAS II, the artificial pupil was set at 4 mm (Güell et al., 2004). Therefore, each subject was instructed to wait in a dark room for 10 min before testing. This measurement was also performed in a dark room, in which the illumination was ~25 lux, to prevent pupil size from influencing the optical quality parameter values. At least three repeated measurements were performed by the same examiner; these repeated measurements were averaged to obtain the mean values of the parameters.

### Visual function

Visual function was assessed by UCVA, CSF and the vision-related questionaires regarding uncomfortable experiences. CSF was measured in a dark room with a commercialized visual function test (Zhishiyuan, JH-P02, Model NO.102JST190828001, Jiangsu Juehua Medical Technology Co., Ltd., China), at a workstation consisting of a display screen, a control PC, and testing software. Stimuli were generated and controlled by an Intel NUC mini-PC (NUC Co., Ltd., China) running the JAVA platform and presented on a GAMMA-corrected ASUS monitor (27 inches, ASUS Computer Company) with a resolution of 2,560 × 1,440, a refresh rate of 60 Hz, and an average brightness of 74.5 cd/m^2^. The PC generated and controlled all stimuli and presented them on the monitor. To reduce the edge effect, a 0.5° Gaussian ramp was added around stimuli.

**Figure 1:**
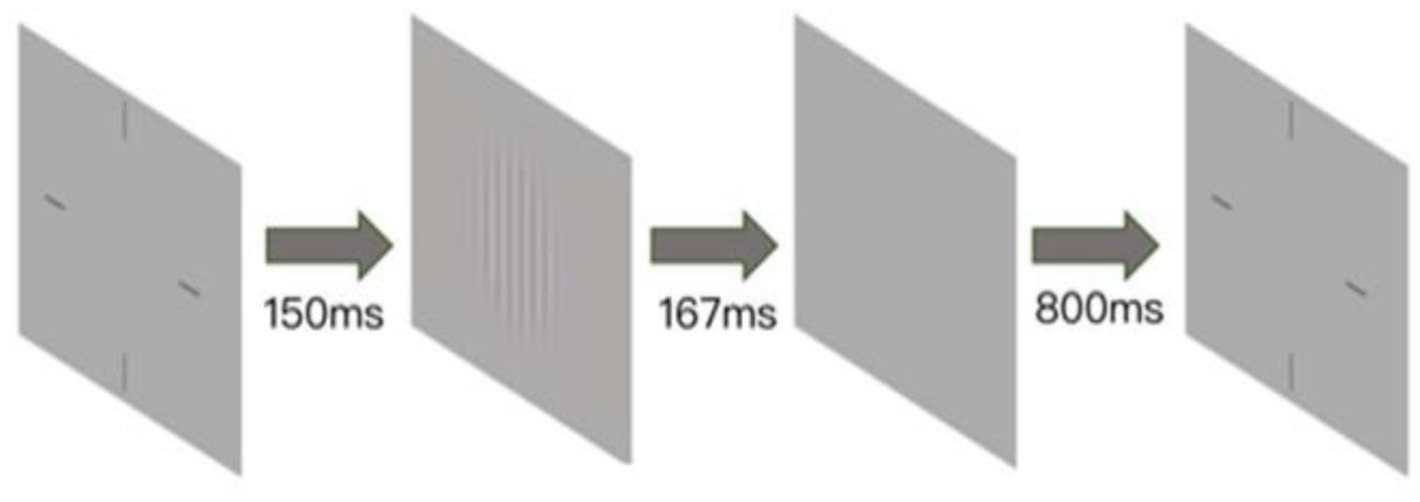
Illustration of contrast sensitivity detection with the Zhishiyuan Testing Station. A brief beep indicates the start of a trial, along with the presentation of a crosshair (3.0 × 3.0°) that indicates the location of the stimulus. After 150 ms, the crosshair disappeared, and a stimulus grating of vertical or horizontal orientation (with equal probability) was displayed for 167 ms. A blank background (mean luminance of 74.5 cd/m^2^) was then displayed, and subjects were instructed to report the orientation of stimuli with the corresponding arrow key. The intertrial interval was 800 ms.

This computerized device has been previously applied in studies concerning CSF after the use of atropine (Cheng et al., 2021). Contrast sensitivity was measured with sinusoidal gratings of spatial frequencies (1.5, 3, 6, 12, 18, and 24 cycles/degree) which were 3.0 × 3.0 at a viewing distance of 2 m. To minimize the edge effect, a 0.5° Gaussian ramp was added to blend the stimulus into the background. Auditory introduction was provided to familiarize subjects with the experiment, stimuli and task conditions before the formal test. There were 270 trials in total, with 45 trials/frequency. Test trials for different frequencies were interleaved. Each trial started with a brief beep and a stimulus-indicating crosshair (3.0 × 3.0). After 150 ms, the crosshair disappeared, and a stimulus grating of either vertical or horizontal orientation (with equal probability) was displayed for 167 ms, followed by a blank background with mean luminance (74.5 cd/m^2^). Subjects were asked to report the orientation with the corresponding arrow key, which also initiated the next trial after an intertrial interval of 800 ms. A Psi method was used to control the grating contrast and tracked contrast threshold that corresponded to 80.3% correct for each spatial frequency (Kontsevich and Tyler, 1999). Contrast sensitivity was calculated as the reciprocal of the contrast threshold. The bit-stealing method was used to achieve high-precision grayscale stimulation (Tyler, 1997). Contrast sensitivity was calculated as the reciprocal of the contrast threshold. The detailed procedures and methods are shown in Figure 1.

The area under the logarithmic contrast sensitivity function (AULCSF) was calculated from the CSF values. The calculation of the AULCSF has been described by Applegate et al. (1998). Similarly, the logarithm of CSF was plotted as a function of log spatial frequency and fitted third-order polynomials to the data. The fitting function between a fixed bound of 1.5 cycles/degree and 18 cycles/degree was integrated to obtain the AULCSF.

### Statistical analysis

All statistical analyses were performed with SPSS software (ver. 26.0; SPSS Inc., Chicago, Illinoi, United States). The normality of variables was assessed with the Kolmogorov–Smirnov test, and variables with a normal distribution are presented as the mean ± standard deviation (SD). All measurements were conducted in both eyes. There was no significant difference in data from the right and left eyes. Therefore, the data from left eyes were randomly selected for analysis. Repeated-measures analysis of variance (RM-ANOVA) was used to compare uncorrected visual acuity, optical quality and visual function among time points, followed by *post hoc* Bonferroni pairwise comparisons if the main effect was significant, and the *p*-value was automatically corrected for multiple pairwise comparisons with the Bonferroni method. The level of statistical significance was set at *p* < 0.05.

## Results

There was no obvious lens decentration (>1 mm based on corneal topography) in any subject during 3 months of OK lens wear. No children complained of transient or long-term discomfort regarding optical quality and visual function (such as glare, holes, distortions, asterism, scieropia, or blurred vision). There was also no significant difference in baseline data (age, sex, visual acuity and refraction) between participants who completed all follow-ups and participants who did not complete the study (all *p* > 0.05).

### Changes in uncorrected visual acuity

Subjects’ UCVA at baseline and 1 month and 3 months after wearing OK lenses were 0.53 ± 0.18, 0.04 ± 0.04 and −0.01 ± 0.05 logMAR, respectively. There was a statistically significant difference in UCVA among baseline and 1-month and 3-month follow-ups (RM-ANOVA: *p* < 0.001). According to multiple comparisons, UCVA was significantly improved after 1 month and 3 months of wearing OK lenses compared to that at baseline (1 month vs. baseline: *p* < 0.001; 3 months vs. baseline: *p* < 0.001). There was no significant difference in UCVA after 1 month and 3 months of lens wear (*p* = 0.062).

### Changes in optical quality parameters

**Figure 2:**
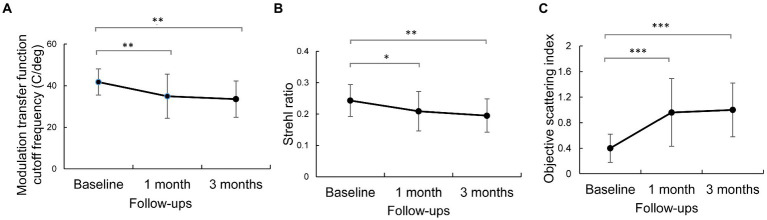
Changes in optical quality parameters after wearing orthokeratology lenses for 3 months. Error bars represent the standard deviation. ***, *p* < 0.001; **, *p* < 0.01 and *, *p* < 0.05, according to *post hoc* Bonferroni tests. **(A)** reprensents the modulation transfer function cut off frequency. **(B)** reprensents strehl ratio. **(C)** reprensents the objective scattering index.

There was a statistically significant difference in MTF cutoff and SR values among the three timepoints (RM-ANOVA: *P*_MTF cutoff_ = 0.004 and *P*_SR_ = 0.014). Compared to baseline, MTF cutoff and SR values were significantly lower after wearing OK lenses (1 month vs. baseline: *P*_MTF_ = 0.008, *P*_SR_ = 0.049; 3 month vs. baseline: *P*_MTF_ = 0.002, *P*_SR_ = 0.003). There was no significant difference in MTF cutoff or SR values at the 1- and 3-month follow-ups (1-month vs. 3-month: *P*_MTF_ = 0.626 and *P*_SR_ = 0.428). The MTF cutoff and SR values stabilized from the 1-month follow-up to the 3-month follow-up (Figures 2A,B).

There was a statistically significant difference in OSI values among baseline and the 1-month and 3-month follow-ups (RM-ANOVA: *P*_OSI_ < 0.001). The OSI values were 0.40 ± 0.22, 0.96 ± 0.53, and 1.00 ± 0.42 at baseline and 1 month and 3 months after wearing OK lenses, respectively. Initial wear of OK lenses significantly increased the OSI values compared to baseline (1-month vs. baseline: *P*_OSI_ < 0.001; 3-month vs. baseline: *P*_OSI_ < 0.001; Figure 2C), whereas the OSI values did not significantly differ between the 3-month and 1-month follow-ups (*P*_OSI_ = 0.720).

**Figure 3:**
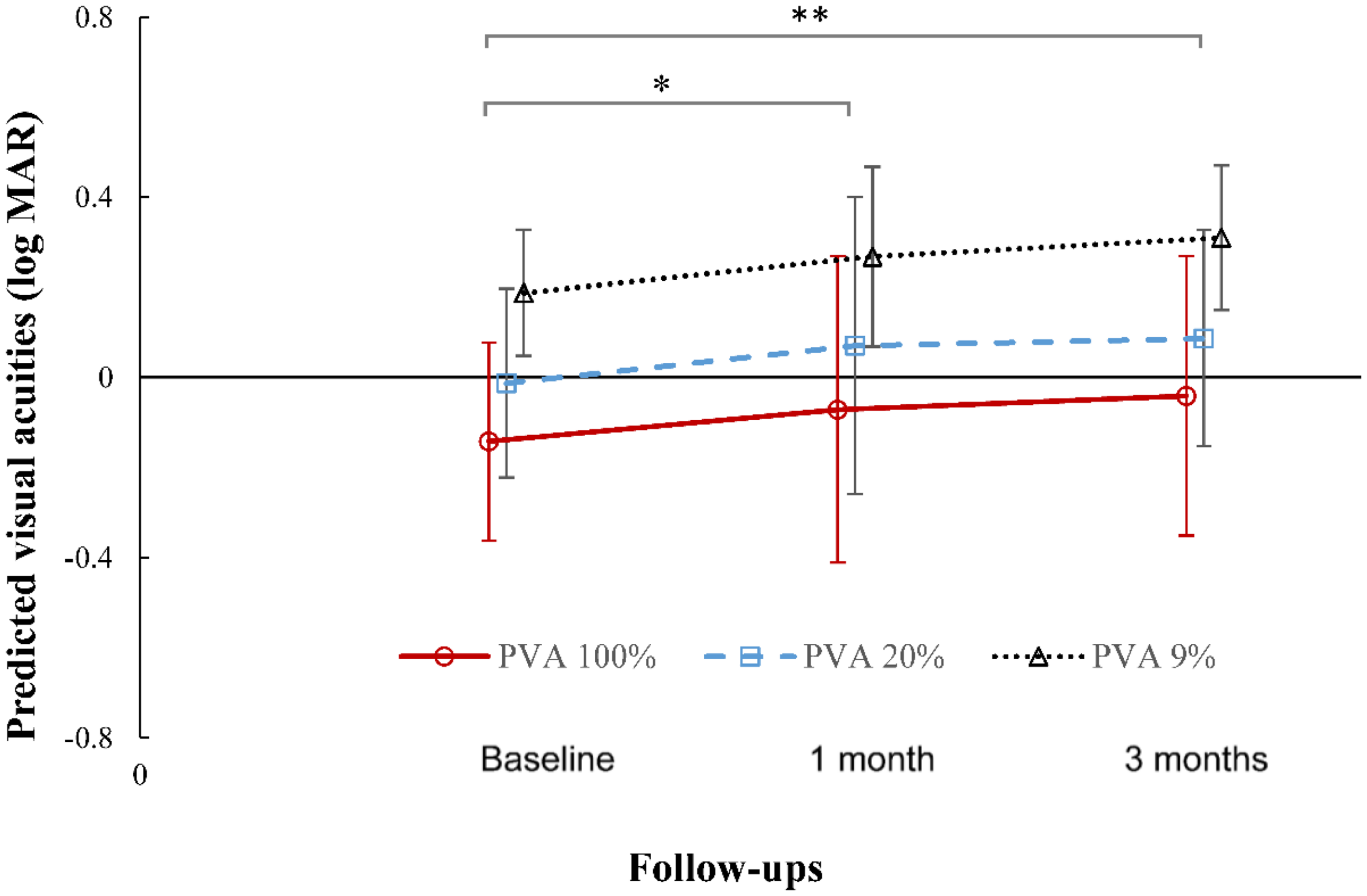
Changes in predicted visual acuity at contrast levels of 100%, 20%, and 9% after wearing orthokeratology lenses for 3 months. Error bars represent the standard deviation. **, *p* < 0.01, significant difference between baseline and 3 months in the predicted visual acuity at contrast levels of 100%, 20%, and 9%. *, *p* < 0.05 between baseline and 1 month in the predicted visual acuity at contrast levels of 100%, 20%, and 9%, according to *post hoc* Bonferroni tests. Small horizontal offsets were added to avoid overlapping error-bars.

After the subjects wore the OK lenses, there was a statistically significant difference in OSI values among baseline and the 1-month and 3-month follow-ups (RM-ANOVA: *P*_PVA100%_ = 0.004, *P*_PVA20%_ = 0.013 and *P*_PVA9%_ = 0.008). According to pairwise multiple comparisons, the predicted visual acuity (PVA) of 100%, 20%, and 9% decreased significantly relative to baseline values (1-month vs. baseline: *P*_PVA100%_ = 0.012, *P*_PVA20%_ = 0.017 and *P*_PVA9%_ = 0.028; 3-month vs. baseline: *P*_PVA100%_ = 0.001, *P*_PVA20%_ = 0.004 and *P*_PVA9%_ = 0.002). There was no significant difference between the 1-month and 3-month values (*P*_PVA100%_ = 0.427, *P*_PVA20%_ = 0.618 and *P*_PVA9%_ = 0.409). The change trend was stable from 1 month to 3 months (Figure 3).

### Changes in visual function parameters

**Figure 4:**
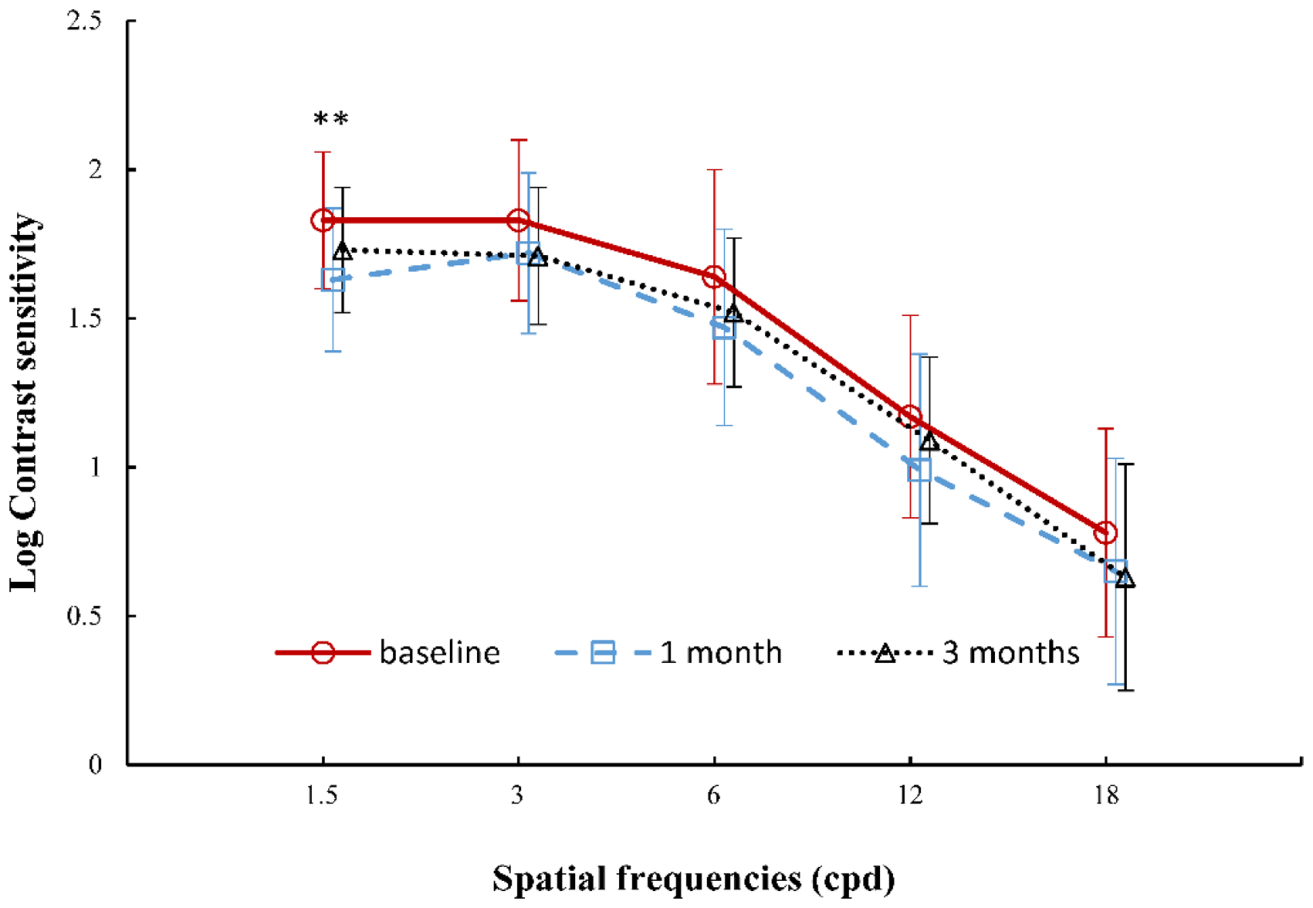
Changes in contrast sensitivity function at different spatial frequencies after wearing orthokeratology lenses for 3 months. Error bars represent the standard deviation. **, *p* < 0.01, significant difference in contrast sensitivity function of 1.5 cpd between baseline and 1 month of wearing the lens, according to *post hoc* Bonferroni tests. Small horizontal offsets were added to avoid overlapping error-bars.

Figure 4 shows the mean monocular contrast sensitivities across five spatial frequencies (1.5, 3, 6, 12, and 18 cpd). There was a significant difference in CSF at 1.5 cpd among baseline and the 1-month and 3-month follow-ups (ANOVA: *P*_1.5 cpd_ = 0.003). Pairwise comparisons showed that CSF at 1.5 cpd decreased significantly after wearing lenses for 1 month compared to that at baseline (*p* = 0.001). After subjects wore the lens for 3 months, the CSF at 1.5 cpd returned to baseline values (3 months vs. baseline: *p* = 0.076). CSF also tended to decrease and then recover at 3, 6, 12, and 18 cpd, but there was no significant difference among baseline and the 1-month and 3-month follow-ups (*P*_3cpd_ = 0.336, *P*_6cpd_ = 0.147, *P*_12cpd_ = 0.131, *P*_18cpd_ = 0.262; Figure 4).

The AULCSF values were 1.67 ± 0.26, 1.51 ± 0.29, and 1.63 ± 0.32 at baseline and at 1 month and 3 months after wearing lenses, respectively. There was no significant difference in AULCSF values among baseline and the 1-month and 3-month follow-ups (ANOVA: *p* = 0.114).

### Changes in corneal topography parameters

There was a statistically significant difference in steep K among baseline, 1-month and 3-month timepoints (RM-ANOVA: *p* < 0.001). According to multiple comparisons, steep K became significantly flatter after 1 month and 3 months of wearing OK lenses compared to baseline (1-month vs. baseline: *p* < 0.001; 3-month vs. baseline: *p* < 0.001). There was no significant difference in steep K between 1 month and 3 months of lens wear (*p* > 0.05). A similar change trend was also observed in flat K.

## Discussion

In this study, optical quality parameters (the OSI, MTF cutoff, SR, and PVA100%, PVA20% and PVA9% values), visual function parameters (UCVA, contrast sensitivities at 1.5, 3, 6, 12, 18 cpd and AULCSF) and corneal topography were assessed at baseline and at 1 month and 3 months of OK lens wear. The results revealed that the optical quality was more susceptible to OK lenses than visual function in children. Optical quality decreased after 1 month of wearing OK lenses; subsequently, values remained stable and did not rebound to the baseline level by 3 months. Regarding visual function, most of the visual function parameters remained at baseline values with prolongation of wearing time, indicating that visual function was not affected by OK lens wear, especially in later stages of treatment.

In terms of optical quality, the MTF cutoff, SR and PVA values (Figures 2, 3) showed a significant decline with OK lens wear; the OSI value increased significantly in the early stage of OK lens wear and then remained stable without recovery with prolongation of wearing time. This indicated persistent deterioration in the optical quality of retinal images. Xie and Wei (2023) and Liu et al. (2019) reported that the OSI increased within the first 3 months of treatment and exhibited only 20% recovery after 1 year of treatment (Liu et al., 2019). Ocular total and corneal high-order aberrations also increased after wearing OK lenses (Guo et al., 2018; Liu et al., 2019; Chang and Cheng, 2020; Xia et al., 2020). These findings in children are consistent with the observations of the current study. However, these previous studies focused on only single parameters of optical quality or had a very short follow-up period. In the present study, optical quality and visual function was comprehensively assessed by uncorrected visual acuity, corneal topography, OQAS^II^ and CSF for multiple parameters with different clinical significance over a longer follow-up period. Overall, these results indicate that OK lenses can increase intraocular scattering and reduce optical quality, with a slow and modest recovery.

In addition, most studies found that during the first month of OK treatment (night wear of OK lenses with a special inverse geometric design), the central corneal curvature was rapidly flattened, mid-peripheral corneal curvature was steepened (Jiang et al., 2021), and corneal surface regularity was increased (Sun et al., 2017). Peripheral corneal thickness increased significantly (Zhang et al., 2023). A similar rapid change in corneal morphology was also found in the current study during the first month. The change trend in optical quality matched the change trend in corneal morphology and biomechanics. This similarity of change trends between optical quality and corneal histology and physiology may indicate a causal relationship, as proposed by Kong et al. (2020) and Stillitano et al. (2007). During the second to third months of OK lens wear, corneal morphology and biomechanics remained relatively stable. Additionally, optical quality remained stable. The deterioration of optical quality may be related to the stability of the tear film (Xie and Wei, 2023), the relative diameter of the pupil and the diameter of the treatment zone, OK lens decentration (Faria-Ribeiro et al., 2016; Liu et al., 2018), and so on. Therefore, the initial month of OK treatment of children is a key period that merits close attention to the effect of these factors on discomfort related to deterioration of optical quality.

Regarding the visual function parameters, UCVA, as central visual acuity under high contrast conditions, improved and remained stable after 1 month of OK lens use. In addition, CSF reflects the visual ability to distinguish an object from the background of a different contrast, which is thought to be a better predictor of visual performance on quality of life. CSF at 3–18 cpd (each CSF, Figure 4) and AULCSF (whole CSF) showed no change after 1 month or 3 months of OK lens wear. Review of the CSF literature revealed that only three studies have been conducted in children. Singh et al. (2020) reported no change after 1 month under dark light conditions with PACT in children. Guo et al. (2018) also found no change at 3–18 cpd under mesopic conditions after wearing lenses for 1 month by CSV-1000, and AULCSF was not significantly changed. In the current study, a similar result was observed in CSF at 3–18 cpd and AULCSF under similar conditions when using a different device (Zhishiyuan). However, Chang and Cheng (2020) found that CSF decreased at 1.5, 3, and 12 cpd under night-vision conditions using stereo optical functional vision analysis. In the current study, only CSF at 1.5 cpd decreased significantly in the early stage of lens wearing (1 month); it recovered after 3 months of lens wear. There was an obvious difference in the examination equipment among these studies, which led to the different results. CSV-1000 and PACT are unable to measure CSF at 1.5 cpd. However, these results both indicate that visual function in low-contrast conditions may be affected to a certain extent. This change trend was also observed in the initial month of treatment. Combined with the rapid decline in optical quality, discomfort at night may occur only during the initial month of treatment. The decline in CSF at 1.5 cpd may be related to dark conditions. A larger pupil diameter leads to more HOA and scattering of light in the eye, resulting in worse CSF. This slight CSF decline could be easily detected under lower contrast night-vision conditions. The reasons for this decline should be explored with more in-depth studies. In other words, for OK lens wearers, the pupil diameter needs to be measured before OK lens wear. OK lenses with a larger optic zone diameter may help to reduce subjective visual discomfort caused by a large pupil diameter, especially for wearers who need to work under night conditions, such as night driving.

Overall, the main finding of this study is that the trends in optical quality and visual function were not consistent after 3 months of OK lens wear. In the initial month of treatment, few visual function parameters were affected by OK lens wear, despite rapid degradation of optical quality. The theory of neural adaptation to optical aberrations and scattering of light proposed by Chen et al. (2007) and Bao and Engel (2012) may explain these inconsistent changes. In addition, Hiraoka et al. (2009) and Guo et al. (2018) both reported that most patients were highly satisfied with OK lenses in the clinic. Chang and Cheng (2020) and Costa et al. (2013) proposed that children may exhibit good neural adaptation that compensates for the decrease in optical quality induced by OK lenses. Guo et al. (2018) found that only three children had transient complaints of light distortion, which were alleviated after a few days. In the current study, no children reported any discomfort. Chang and Cheng (2020) proposed that children may exhibit better neural adaptation that compensates for optical aberrations induced by OK lenses after comparing CSF results of children and adults. Therefore, older wearers should pay more attention to optical quality and visual function, especially during the first month of OK lens use. Based on the theory of neural adaptation, in later stages, visual function is less susceptible to a decline induced by OK lenses.

## Data availability statement

The raw data supporting the conclusions of this article will be made available by the authors, without undue reservation.

## Ethics statement

The studies involving human participants were reviewed and approved by the Ethics Committee of the Eye Hospital of Wenzhou Medical University. Written informed consent to participate in this study was provided by the participants' legal guardian/next of kin.

## Author contributions

WL, FL, and WJ conceived the experiments. GS, YuZ, YL, and KM performed the experiments. QL, YJ, and YaZ analyzed and interpreted the data. WL and GS wrote the manuscript. All authors contributed to manuscript revision and read and approved the submitted version.

## Funding

This study was supported by the Innovation and Guidance project of Eye Hospital affiliated to Wenzhou Medical University (YNCX3201907) and the Wenzhou Science and Technology Bureau Basic Projects (grant nos. Y2020348, Y20210203, and Y20190630). The sponsors or funding agencies had no role in the design or implementation of this research.

## Conflict of interest

The authors declare that the research was conducted in the absence of any commercial or financial relationships that could be construed as a potential conflict of interest.

## Publisher’s note

All claims expressed in this article are solely those of the authors and do not necessarily represent those of their affiliated organizations, or those of the publisher, the editors and the reviewers. Any product that may be evaluated in this article, or claim that may be made by its manufacturer, is not guaranteed or endorsed by the publisher.

## Footnotes

^1^ http://www.chictr.org.cn/index.aspx
